# The Joint Effect of Renal Function Status and Coagulation Biomarkers on In‐Hospital Outcomes in Acute Ischemic Stroke Patients With Intravenous Thrombolysis

**DOI:** 10.1002/iid3.70099

**Published:** 2024-12-11

**Authors:** Manli Lu, Junwen Xue, Yi Wang, Dongqin Chen, Yongjun Cao, Chongke Zhong, Xia Zhang

**Affiliations:** ^1^ Department of Neurology and Clinical Research Center of Neurological Disease The Second Affiliated Hospital of Soochow University Suzhou Jiangsu China; ^2^ Department of Epidemiology School of Public Health, Medical College of Soochow University Suzhou Jiangsu China

**Keywords:** acute ischemic stroke, d‐dimer, fibrinogen, glomerular filtration rate, joint effect, thrombolytic therapy

## Abstract

**Objective:**

To demonstrate whether combining renal function status [estimating glomerular filtration rate (eGFR)] with coagulation biomarkers [fibrinogen (Fg) and d‐dimer] is more beneficial in predicting in‐hospital outcomes following intravenous thrombolysis (IVT) in acute ischemic stroke (AIS) patients.

**Methods:**

We studied 417 AIS patients with IVT. According to the cut‐offs of coagulation biomarkers (Fg and d‐dimer) and eGFR determined by receiver operating characteristic (ROC) curves, the patients were divided into four groups: LFLG (low Fg and low eGFR), LFHG (low Fg and high eGFR), HFLG (high Fg and low eGFR), and HFHG (high Fg and high eGFR); or LDLG (low d‐dimer and low eGFR), LDHG (low d‐dimer and high eGFR), HDLG (high d‐dimer and low eGFR), and HDHG (high d‐dimer and high eGFR). Logistic regression models were used to calculate odds ratios (ORs) and 95% confidence intervals (CIs) for poor outcomes at discharge and post‐stroke pneumonia across the four groups.

**Results:**

The patients in the HFLG and HDLG groups had the poorest prognosis at discharge and the highest risk of in‐hospital pneumonia. They experienced 3.00 or 4.59 times higher risk of in‐hospital pneumonia than those in the LFHG and LDHG groups (95%CI: 1.07–8.44, *p* < 0.05; 95%CI: 1.58–13.32, *p* = 0.005). Similarly, the risk of adverse outcome at discharge was 3.02 and 1.52 times higher in HFLG and HDLG groups (95%CI: 1.63–9.91, *p* < 0.005; 95%CI: 1.11–5.74, *p* < 0.05) compared to that in LFHG and LDHG groups. Adding eGFR and Fg or d‐dimer to the risk model improved the risk reclassification for in‐hospital pneumonia and functional outcomes at discharge.

**Conclusion:**

Combining renal function status and coagulation biomarkers within 4.5 h after onset could better predict in‐hospital outcomes of AIS patients with IVT.

## Graphical abstract

Combining renal function status and coagulation biomarkers Within 4.5 h after onset could better predict in‐hospital outcomes of AIS patients With IVT. This provides a simple and useful tool for the identification of high‐risk patients and allows for better individualized and accurate treatment.

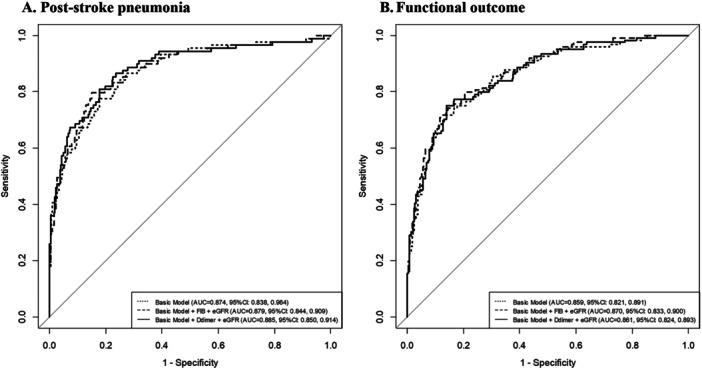

## 1 Introduction

Ischemic stroke develops due to the interruption of blood supply to the brain. Intravenous thrombolysis (IVT) is one of the two most effective revascularization therapies for acute ischemic stroke (AIS). Impaired renal function and coagulation disorders are frequently found in AIS patients [1], especially in patients with IVT [2, 3].

Estimating the glomerular filtration rate (eGFR) is an important indicator of renal function, which represents the amount of plasma passing through the glomerulus per unit time [4]. Several studies reported that reduced eGFR was associated with more complications, higher mortality rates, and poorer prognosis during post‐stroke recovery [5, 6, 7, 8]. In thrombolytic patients, eGFR was identified as an independent predictor of intracranial hemorrhage (ICH), in‐hospital mortality, and poor functional outcomes at 3 months [9, 10]. Moreover, the impairment of endogenous tissue fibrinogen activator release due to chronic kidney dysfunction (CKD) increased the levels of lipoprotein(a) and plasminogen activator inhibitor‐1, further inhibiting plasminogen activation [10, 11]. This inhibition can compromise the reperfusion effects of IVT [12].

Additionally, Fg and d‐dimer levels have been shown to independently predict the risk of stroke‐associated pneumonia (SAP) [13], cognitive impairment [14], and clinical outcomes [15] in AIS patients. The close correlation among thrombosis, inflammation, and renal function status indicates a combined effect of renal function status and coagulation biomarkers on AIS outcomes based on similar mechanisms. However, studies examining the combined role of renal function status and coagulation biomarkers in predicting stroke outcomes, especially in patients with IVT, are lacking.

Up to one‐third of individuals who have suffered a stroke experience pneumonia after stroke [16, 17], which is associated with increased in‐hospital mortality, prolonged hospital stay length, poor functional outcomes, and a considerable economic impact on healthcare resources [18, 19]. Besides, pneumonia is the main manifestation of inflammation. Therefore, in this study, we chose functional outcomes at discharge and in‐hospital pneumonia as endpoints. We aimed to explore the combined value of eGFR and coagulation biomarkers in predicting in‐hospital outcomes, including functional outcomes at discharge and in‐hospital pneumonia, in AIS patients undergoing IVT.

## 2 Methods

### 2.1 Study Participants

We retrospectively and consecutively screened a total of 496 AIS patients in the emergency department (ED) from July 2018 to August 2021 at the Second Affiliated Hospital of Soochow University. Patients older than 18 years with a confirmed diagnosis of AIS by cranial CT or MRI were included. All participants were admitted to the hospital within 4.5 h after onset and received rt‐PA thrombolytic therapy with informed consent.

Patients with cerebral infarction sequelae and a modified Rankin Scale (mRS) score of ≥ 1 were excluded. Severe underlying conditions, such as autoimmune diseases, chronic inflammatory diseases, severe hepatic, renal, cardiac, or respiratory diseases, and known malignancy were considered ineligible. Additional exclusion criteria were as follows [1]: pregnancy [2], an infection within 1 week before admission [3], receiving inpatient treatment in other hospitals [4], diagnosis of TIA based on completely revised symptoms, and no acute infarct on the MRI or follow‐up CT scans [5], and lack of data on admission renal function and coagulation biomarker testing.

**Figure 1:**
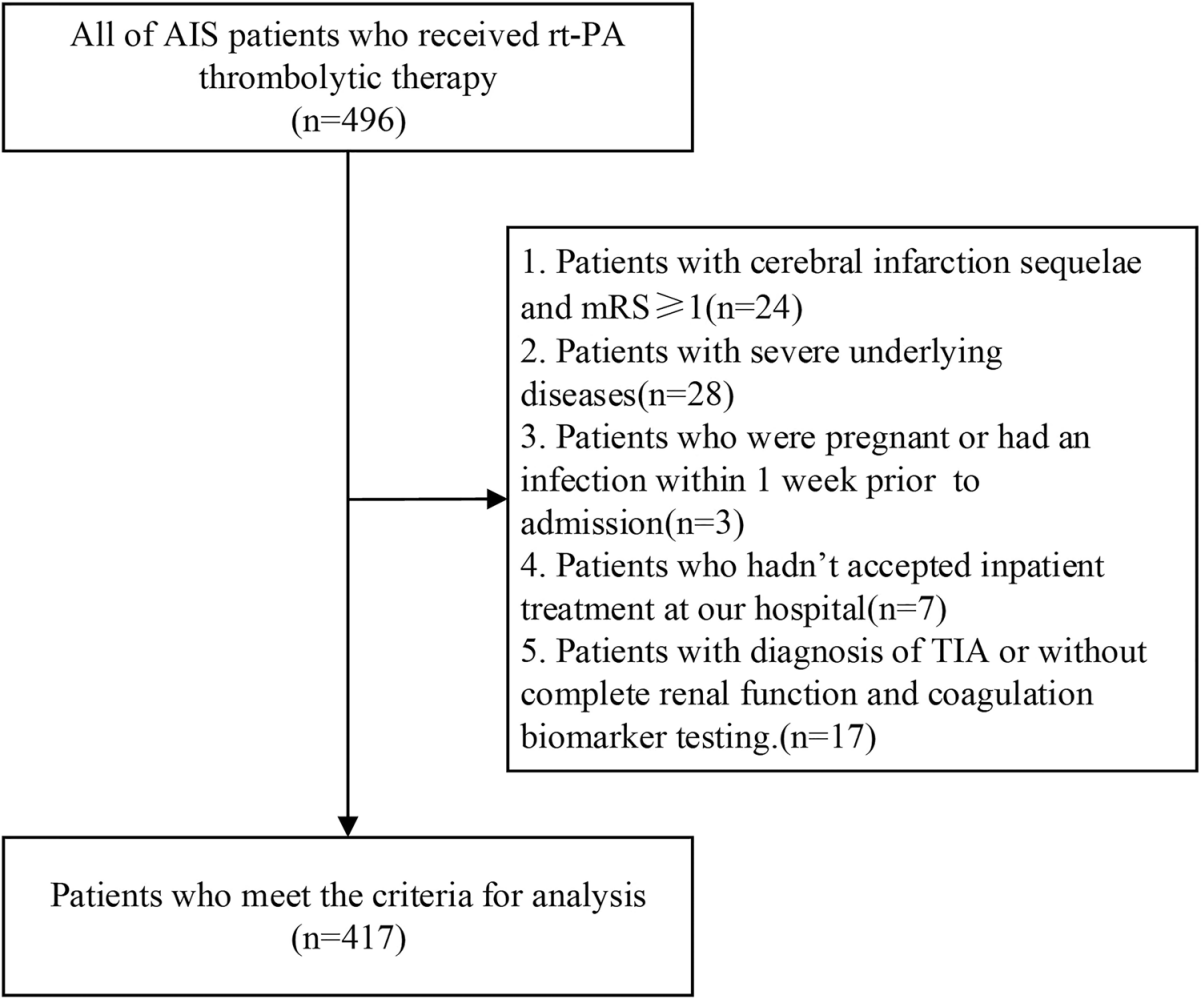
Patient flowchart. AIS, acute ischemic stroke; mRS, modified Rankin Scale.

Finally, 417 patients were included in the analysis (Figure 1). The study was approved by the Ethics Committee of the Second Affiliated Hospital of Soochow University, China (JDHG‐2021‐41).

### 2.2 Data Collection and Outcome Measures

Demographics, vascular risk factors, stroke subtypes, great vessel occlusion, and duration of hospitalization were standardly collected at the enrollment. Vascular risk factors such as hypertension, hyperglycemia, hyperlipidemia, coronary heart disease, previous stroke history, and cigarette smoking were ascertained by standard criteria. Previous medication history, including anticoagulants or antiplatelet drugs, was also recorded. Stroke subtypes fulfilling ASCO classification criteria included A for atherosclerosis, S for small vessel disease, C for cardiac source, and O for other causes. Stroke symptom was defined by the Oxfordshire Community Stroke Project (OCSP) classification, including lacunar infarct (LACI), total anterior circulation infarcts (TACI), partial anterior circulation infarcts (PACI), and posterior circulation infarcts (POCI). Renal function and coagulation biomarkers were measured before IVT in the ED using the VITROS XT 3400 Chemistry Analyzer and the Sysmex CS‐5100 Coagulation Analyzer.

National Institutes of Health Stroke Scale (NIHSS) scores were assessed by trained neurologists at admission. Physical function at discharge was evaluated by the modified Rankin Scale (mRS). A good prognosis at discharge was defined as an mRS of 0 to 2. In‐hospital pneumonia was diagnosed by treating physicians in terms of the criteria of the Center for Disease Control and Prevention for hospital‐acquired pneumonia. Patients should present clinical symptoms such as chest pain during breathing or coughing, altered consciousness, cough, fatigue, fever or chills, nausea, vomiting, or diarrhea. Additionally, there should be positive laboratory biomarkers of inflammation, along with new or progressive chest CT findings, including infiltrates, consolidation, or ground‐glass opacities. All‐cause in‐hospital mortality was recorded.

GFR was estimated using the Chronic Kidney Disease Epidemiology Collaboration equation [20] (CKD‐EPI): GFR = α × (Scr/κ)^υ^ × (0.993)^Age^, which takes into account the patient's age, gender, race, and serum creatinine level. The result is reported in units of mL/min/1.73 m^2^.

### 2.3 Statistical Analysis

SAS statistical software (version 9.4; SAS Institute, Cary, NC) was used for statistical description and analysis. Continuous variables of baseline characteristics are expressed as mean ± SD or median interquartile interval (IQR), and compared by Student's *t*‐test or Wilcoxon rank‐sum test among the groups. The categorical variables are expressed as percentages and compared using a *χ* ^2^ test.

The joint effects of eGFR and coagulation biomarkers (Fg or d‐dimer) on the risk of post‐stroke pneumonia and functional outcome were further investigated. The participants were divided into four groups according to the cut‐points of eGFR and coagulation biomarkers (Fg or d‐dimer) levels determined by the receiver operating characteristic (ROC) curves as LFLG (low Fg and low eGFR), LFHG (low Fg and high eGFR), HFLG (high Fg and low eGFR), and HFHG (high Fg and high eGFR); or LDLG (low d‐dimer and low eGFR), LDHG (low d‐dimer and high eGFR), HDLG (high d‐dimer and low eGFR), and HDHG (high d‐dimer and high eGFR). Univariate and multivariate logistic regression models were used to calculate the odds ratios (ORs) and 95% confidence intervals (CIs) of hospitalization outcomes of thrombolytic patients among four groups. Since these factors such as age, sex, smoking history, admission NIHSS score, vascular history (hypertension, hyperglycemia, hyperlipidemia, and coronary heart disease), previous stroke history, ischemic stroke syndrome, as well as the use of anticoagulant and antiplatelet medications and hospitalization duration, have been established in the literature as significantly related to stroke risk and outcomes [21, 22]. All the above factors were included in the multivariate analysis. Then, to evaluate the predictive significance of joint effects of renal function status and coagulation biomarkers on clinical outcomes, C statistics, the net reclassification index (NRI), and the integrated discrimination improvement (IDI) were calculated by adding eGFR and Fg or d‐dimer to the model with established risk factors. All *p‐*values were two‐tailed, and a significance level of 0.05 was applied.

## 3 Results

A total of 417 patients were enrolled, with 239 men (57.3%) and a mean age(SD) of 67.8(13.2) years. The mean baseline NIHSS score was 4 [2, 3, 4, 5, 6, 7, 8, 9, 10]. There was no in‐hospital death. Compared with non‐pneumonia patients, patients developing pneumonia during hospitalization were older with longer hospital stays and higher NIHSS scores at admission (all *p* < 0.05). They also had higher proportions of great vessel occlusion, small‐artery occlusion, and TACI subtypes and lower proportions of atherosclerosis and PACI subtypes (all *p* < 0.05). In comparison to patients with favorable at‐discharge functional outcomes, those with adverse outcomes were older and had more females, longer hospital stays, and higher NIHSS scores at admission, with higher proportions of large artery occlusion, small‐artery occlusion, TACI subtype, and hypertension history. The proportions of patients with atherosclerosis, cardioembolism, and PACI and LACI subtypes were lower (all *p* < 0.05) (Table 1).

**Table 1:** Baseline characteristics of study participants according to post‐stroke pneumonia and at‐discharge functional outcome.

	Post‐stroke pneumonia			Functional outcome		
Characteristics^a^	Non‐pneumonia	Pneumonia	*p* value	Good	Poor	*p* value
No. of patients	328	89		293	124	
Age, y	66.5 ± 12.9	72.9 ± 13.2	< 0.001	66.2 ± 13.0	71.7 ± 13.0	< 0.001
Male sex	193 (58.8)	46 (51.7)	0.23	179 (61.1)	60 (48.4)	0.02
Current cigarette smoking status	119 (36.3)	27 (30.3)	0.30	111 (37.9)	35 (28.2)	0.06
Admission NIHSS score	3 (2–6)	12 (7‐20)	< 0.001	3 (2–5)	11 (6–16)	< 0.001
Great vessels	45 (13.7)	54 (60.7)	< 0.001	32 (10.9)	67(54.0)	< 0.001
Medical history						
Hypertension	210 (64.0)	62 (69.7)	0.32	181 (61.8)	91 (73.4)	0.02
Hyperglycemia	69 (21.0)	18 (20.2)	0.87	54 (18.4)	33 (26.6)	0.06
Hyperlipidemia	68 (20.7)	15 (16.9)	0.42	62 (21.2)	21 (16.9)	0.32
Coronary heart disease	21 (6.4)	6 (6.7)	0.91	18 (6.1)	9 (7.3)	0.67
History of stroke	44 (13.5)	13 (14.6)	0.78	42 (14.4)	15 (12.1)	0.57
Medications						
Anticoagulant	6 (1.8)	1 (1.1)	0.65	4 (1.4)	3 (2.4)	0.44
Antiplatelet	15 (4.6)	4 (4.5)	0.97	13 (4.4)	6 (4.8)	0.86
ACSO						
A	256 (78.0)	48 (53.9)	< 0.001	230 (78.5)	74 (59.7)	< 0.001
C	18 (5.5)	3 (3.4)	0.42	19 (6.5)	2 (1.6)	0.04
S	48 (14.6)	37 (41.6)	< 0.001	40 (13.7)	45 (36.3)	< 0.001
O	6 (1.8)	1 (1.1)	1.00	4 (1.4)	3 (2.4)	0.44
Stroke syndrome						
TACI	37 (11.3)	42 (47.2)	< 0.001	27 (9.2)	52 (41.9)	< 0.001
PACI	217 (66.2)	36 (40.4)	< 0.001	199 (67.9)	54 (43.5)	< 0.001
POCI	63 (19.2)	11 (12.4)	0.13	56 (19.1)	18 (14.5)	0.26
LACI	11 (3.4)	0	0.08	11 (3.8)	0	0.04
Duration of hospitalization	8 (7‐10)	13 (8‐19)	<0.001	8 (7‐10)	11(8‐16)	<0.001

^a^ Continuous variables are expressed as mean ± standard deviation or median (interquartile range). Categorical variables are expressed as number (%).

Abbreviations: A, atherosclerosis; C, cardiac source; LACI, lacunar infarcts; NIHSS, National Institute of Health Stroke Scale; O, other causes; PACI, partial anterior circulation infarcts; POCI, posterior circulation infarcts; S, small vessel disease; TACI, total anterior circulation infarcts.

### 3.1 Single and Combined Predictive Role of eGFR and Fg in Post‐Stroke Pneumonia

These results are given in Table 2. A total of 89(21.3%) patients had in‐hospital pneumonia. Grouped by cut‐offs of eGFR or Fg according to ROC curves, univariate analysis showed that patients with low eGFR or high Fg levels had a significantly higher risk of in‐hospital pneumonia (all *p* < 0.005). After adjusting for multiple variables, the correlation of Fg still existed (*p* < 0.05). Patients with high Fg levels were 2.19 times more likely to develop in‐hospital pneumonia (Table 2).

**Table 2:** Separate and joint effects of eGFR and Fg on post‐stroke pneumonia and at‐discharge functional outcome of AIS patients with thrombolysis.

		Unadjusted			Multivariable adjusted		
	Events, %	OR	95% CI	*p* value	OR	95% CI	*p* value
Post‐stroke pneumonia							
Low eGFR (< 90)	60 (27.5)	2.23	1.36–3.65	0.002	1.40	0.61–3.21	0.43
High Fg (> 3.18)	43 (33.1)	2.59	1.60–4.20	< 0.001	2.19	1.16–4.12	0.02
Joint effect of Fg and eGFR							
Fg (‐) and eGFR (‐)	19 (13.0)	1.00			1.00		
Fg (‐) and eGFR (+)	27 (19.1)	1.58	0.84–3.00	0.16	1.04	0.39–2.78	0.93
Fg (+) and eGFR (‐)	10 (18.9)	1.55	0.67–3.60	0.30	1.35	0.46–3.93	0.58
Fg (+) and eGFR (+)	33 (42.9)	5.01	2.59–9.70	< 0.001	3.00	1.07‐8.44	0.04
Functional outcome							
Low eGFR (< 90)	83 (38.1)	2.37	1.53–3.67	< 0.001	1.96	0.97–3.97	0.06
High Fg (> 2.91)	77 (40.1)	2.54	1.65–3.91	< 0.001	2.26	1.31–3.90	0.004
Joint effect of Fg and eGFR							
Fg (‐) and eGFR (‐)	18 (15.9)	1.00			1.00		
Fg (‐) and eGFR (+)	29 (25.9)	1.84	0.956–3.56	0.07	1.34	0.52–3.51	0.55
Fg (+) and eGFR (‐)	23 (26.7)	1.93	0.96–3.86	0.06	1.55	0.65–3.68	0.32
Fg (+) and eGFR (+)	54 (50.9)	5.48	2.91–10.31	< 0.001	4.02	1.63–9.91	0.003

*Note:* Data are Odds ratios (95% confidence intervals). Multivariable adjusted including age, sex, current smoking status, admission NIHSS score, great vessels, medical history (hypertension, hyperglycemia, hyperlipidemia, and coronary heart disease), history of stroke, ischemic stroke syndrome, use of anticoagulant and antiplatelet medication, and duration of hospitalization.

Abbreviations: CI, confidence interval; eGFR, estimating glomerular filtration rate; Fg, Fibrinogen; OR, odds ratios.

Moreover, patients in the HFLG group had the highest incidence of post‐stroke pneumonia. Compared to the LFHG group, the unadjusted OR for patients in the HFLG group was 5.01(95% CI: 2.59–9.70) for post‐stroke pneumonia. After adjusting for age, sex, current smoking status, admission NIHSS score, great vessel occlusion, medical history (including hypertension, hyperglycemia, hyperlipidemia, and coronary heart disease), previous stroke history, ischemic stroke syndrome, and the use of anticoagulant and antiplatelet medications, the adjusted OR for the HFLG group for post‐stroke pneumonia was 3.00 (95% CI: 1.07–8.44) compared to the LFHG group (Table 2).

### 3.2 Single and Combined Predictive Role of eGFR and Fg in Functional Outcome At‐Discharge

These results are given in Table 2. A total of 124(29.7%) participants experienced adverse functional outcomes at discharge. Similarly, patients with low eGFR or high Fg levels showed a higher risk of adverse outcomes revealed by univariate analysis (all *p* < 0.001). After adjusting for several variables, high Fg levels were still proven to be strongly correlated with poor prognosis. The multivariable‐adjusted OR (95%CI) for patients with high Fg levels was 2.26(1.31–3.90) for poor functional outcomes at discharge (Table 2).

Additionally, those in the HFLG group had the highest risk of poor functional outcomes at discharge. Patients in the HFLG group were 5.48 times more likely to experience poor functional outcomes than those in the LFHG group. After adjusting for the aforementioned variables, the risk remained 4.02 times higher (95% CI: 1.63–9.91, *p* < 0.005) (Table 2).

### 3.3 Single and Combined Predictive Role of eGFR and d‐Dimer in Post‐Stroke Pneumonia

These results are given in Table 3. Univariate analysis revealed that high d‐dimer levels were associated with an increased risk of in‐hospital pneumonia (*p* < 0.001). High d‐dimer levels were still proven to be tightly associated with a higher risk of in‐hospital pneumonia after adjusting for several variables. The OR (95% CI) of high d‐dimer levels for in‐hospital pneumonia was 3.34 (1.75–6.39) after multivariable adjustment (Table 3).

**Table 3:** Separate and joint effects of eGFR and d‐dimer on post‐stroke pneumonia and at‐discharge functional outcome of AIS patients with thrombolysis.

		Unadjusted			Multivariable adjusted		
	Events, %	OR	95% CI	*P* value	OR	95% CI	*P* value
Post‐stroke pneumonia							
Low eGFR (< 90)	60 (27.5)	2.23	1.36–3.65	0.002	1.40	0.61–3.21	0.43
High d‐dimer (> 1.32)	53 (41.7)	5.05	3.08–8.30	< 0.001	3.34	1.75–6.39	< 0.001
Joint effect of d‐dimer and eGFR							
d‐dimer (‐) and eGFR (‐)	13 (8.1)	1.00			1.00		
d‐dimer (‐) and eGFR (+)	23 (17.8)	2.47	1.20–5.10	0.01	1.49	0.53–4.17	0.44
d‐dimer (+) and eGFR (‐)	16 (42.1)	8.28	3.51–19.53	< 0.001	3.84	1.27–11.58	0.02
d‐dimer (+) and eGFR (+)	37 (41.6)	8.10	4.00–16.42	< 0.001	4.59	1.58–13.32	0.005
Functional outcome							
Low eGFR (< 90)	83 (38.1)	2.37	1.53–3.67	< 0.001	1.96	0.97–3.97	0.06
High d‐dimer (> 1.08)	67 (42.4)	2.61	1.70–4.02	< 0.001	1.27	0.71–2.26	0.42
Joint effect of d‐dimer and eGFR							
d‐dimer (‐) and eGFR (‐)	26 (16.8)	1.00			1.00		
d‐dimer (‐) and eGFR (+)	31 (29.8)	2.11	1.16–3.82	0.01	2.03	0.91–4.52	0.08
d‐dimer (+) and eGFR (‐)	15 (34.1)	5.57	1.21–5.45	0.01	1.20	0.48–2.97	0.70
d‐dimer (+) and eGFR (+)	52 (45.6)	4.16	2.38–7.28	< 0.001	2.52	1.11–5.74	0.03

*Note:* Data are Odds ratios (95% confidence intervals). Multivariable adjusted including age, sex, current smoking status, admission NIHSS score, great vessels, medical history (hypertension, hyperglycemia, hyperlipidemia, and coronary heart disease), history of stroke, ischemic stroke syndrome, use of anticoagulant and antiplatelet medication, and duration of hospitalization.

Abbreviations: CI, confidence interval; eGFR, estimating glomerular filtration rate; Fg, Fibrinogen; OR, odds ratios.

Moreover, thrombolytic patients in the HDLG group were most likely to experience in‐hospital pneumonia. Patients in the HDLG group were 8.10 times more likely to experience pneumonia than those in the LDHG group (95% CI: 4.00–16.42, *p* < 0.001). This risk remained 4.59 times higher after adjusting for age, baseline NIHSS score, great vessel occlusion, and other traditional risk factors (95% CI 1.58–13.32, *p* < 0.005) (Table 3).

### 3.4 Single and Combined Predictive Role of eGFR and d‐Dimer in At‐Discharge Functional Outcome

These results are given in Table 3. Univariate analysis demonstrated that high d‐dimer levels were correlated with poor functional outcomes at discharge(*p* < 0.001), which became insignificant after adjusting several factors(*p* > 0.05).

Additionally, compared to the LDHG group, the unadjusted OR for poor outcome at discharge in the HDLG group was 4.16 (95% CI 2.38–7.28). After adjusting for the aforementioned confounders, the OR(95%) remained significant at 2.52(1.11–5.74) (Table 3).

### 3.5 Predictive Performance of the Model Combined With eGFR and Coagulation Biomarkers

**Figure 2:**
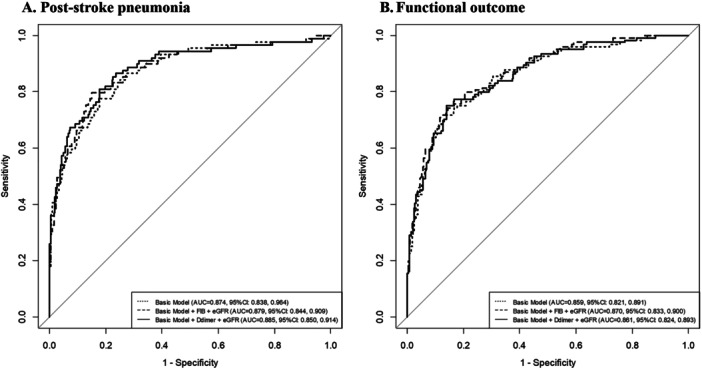
(A). ROC curves of combined coagulation biomarkers (Fg or d‐dimer) and estimating glomerular filtration rate (eGFR) levels on post‐stroke pneumonia. (B). ROC curves of combined coagulation biomarkers (Fg or d‐dimer) and estimating glomerular filtration rate (eGFR) levels on at‐discharge functional outcome. AUC, area under ROC curve; eGFR, estimating glomerular filtration rate; Fg, Fibrinogen; ROC, receiver operating characteristic.

These results are given in Table 4 and Figure 2. The basic model included age, sex, current smoking status, admission NIHSS score, great vessel occlusion, medical history (hypertension, hyperglycemia, hyperlipidemia, and coronary heart disease), history of stroke, ischemic stroke syndrome, use of anticoagulant and antiplatelet medication, and duration of hospitalization. When we added eGFR and Fg or d‐dimer to the basic model, C statistics improved from 0.874(95%CI: 0.838–0.964) to 0.879(95%CI: 0.844–0.909) or 0.885(95%CI: 0.850–0.914) for in‐hospital pneumonia; and C statistics improved from 0.859(95%CI: 0.821–0.891) to 0.870(95%CI: 0.833–0.900) or 0.861(95%CI: 0.824–0.893) for functional outcome at discharge (Figure 2).

**Table 4:** Performance of models.

	NRI (category free)		IDI		Calibration	
	Estimate (95% CI), %	*p* value	Estimate (95% CI), %	*p* value	χ^2^ value	*p* value
Post‐stroke Pneumonia						
Basic model	Reference		Reference		9.14	0.33
Basic model + Fg + eGFR	41.2 (18.2–64.2)	< 0.001	1.6 (0.03–3.2)	0.046	7.38	0.50
Basic model + d‐dimer + eGFR	70.6 (48.2–93.0)	< 0.001	3.5 (1.2–5.8)	0.003	11.46	0.18
Functional outcome						
Basic model	Reference		Reference		9.79	0.28
Basic model + Fg + eGFR	40.5 (19.9–61.2)	< 0.001	2.5 (0.9–4.1)	0.002	6.52	0.59
Basic model + d‐dimer + eGFR	27.4 (6.8–48.0)	0.01	0.9 (0.1–1.8)	0.03	8.15	0.42

*Note:* Basic model included age, sex, current smoking status, admission NIHSS score, great vessels, medical history (hypertension, hyperglycemia, hyperlipidemia, and coronary heart disease), history of stroke, ischemic stroke syndrome, use of anticoagulant and antiplatelet medication, duration of hospitalization.

Abbreviations: CI, confidence interval; eGFR, estimating glomerular filtration rate; Fg, Fibrinogen; IDI, integrated discrimination improvement; NRI, net reclassification index.

Furthermore, adding eGFR and Fg or d‐dimer to the basic risk factor model significantly improved the risk reclassification for in‐hospital pneumonia and at‐discharge functional outcome, represented by increased NRI and IDI values (*p* < 0.05) (Table 4). Moreover, the Hosmer–Lemeshow test showed that the models were better calibrated in predicting in‐hospital pneumonia and functional outcome at discharge after the addition of eGFR and coagulation biomarkers (*p* > 0.05).

### 3.6 HFLG or HDLG had the Worst Functional Outcome at Discharge

**Figure 3:**
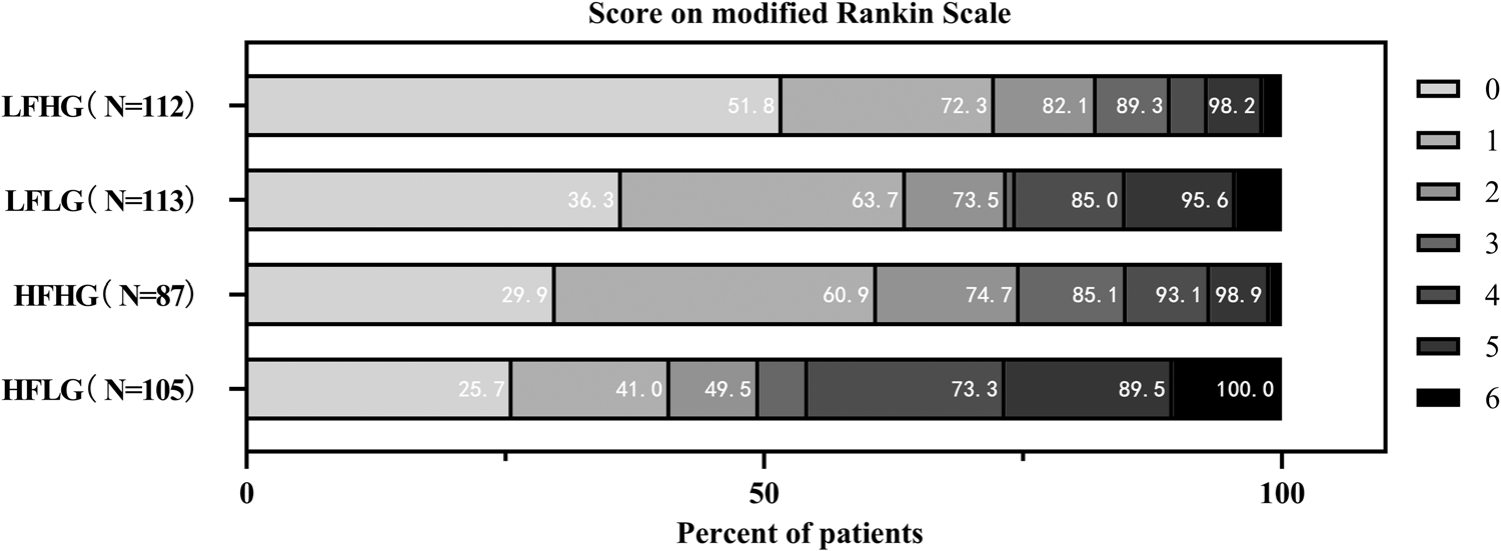
Relationship between mRS at discharge and combined Fg and eGFR levels. eGFR, estimating glomerular filtration rate; Fg, Fibrinogen; HFHG, high Fg and high eGFR; HFLG, high Fg and low eGFR; LFHG, low Fg and high eGFR; LFLG, low Fg and low eGFR; mRS, modified Rankin Scale.

**Figure 4:**
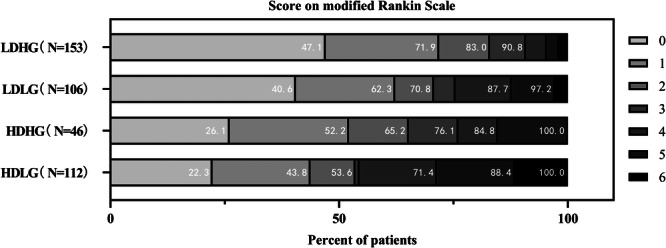
Relationship between mRS at discharge and combined d‐dimer and eGFR levels. eGFR, estimating glomerular filtration rate; HDHG, high d‐dimer and high eGFR; HDLG, high d‐dimer and low eGFR; LDHG, low d‐dimer and high eGFR; LDLG, low d‐dimer and low eGFR; mRS, modified Rankin Scale.

These results are given in Figures 3 and 4. The median mRS score at the discharge of HFLG patients was 3 (IQR 0–5), which was higher compared to that of LFHG patients 0 (IQR 0–2) (Figure 3). Also, HDLG patients had a higher median mRS score of 2 (IQR 1–5) than LDHG patients [1 (IQR 0–2)] (Figure 4).

HFLG and HDLG participants experienced a considerably greater risk of poor outcomes at discharge than those in the LFHG and LDHG groups.

## 4 Discussion

Through the analysis of combined renal function status and coagulation biomarkers at admission with in‐hospital outcomes of 417 AIS patients with IVT, we have made several important findings. First, combined low eGFR and high Fg or d‐dimer levels indicated a high risk of developing post‐stroke pneumonia and a worse at‐discharge outcome. The risk of post‐stroke pneumonia was 3.00 and 4.59 fold in HFLG and HDLG groups in comparison to that in LFHG and LDHG groups. Moreover, compared with patients in the LFHG and LDHG groups, those in the HFLG and HDLG groups were 4.02 and 2.52 times more likely to experience poor at‐discharge outcomes. Second, combining renal function status with coagulation biomarkers enhanced the predictive ability of post‐stroke pneumonia and at‐discharge outcomes of AIS patients with IVT, which provided important guidance for prognostic risk stratification of AIS patients to develop more effective and individual therapeutic regimens.

Several studies have confirmed the association between reduced kidney function and the risk of infections including pneumonia in the general population [23, 24, 25]. For stroke patients, pneumonia is a common complication after stroke [16, 17]. Vart P. et al reported a “U”‐ shaped curve of the association between eGFR and in‐hospital pneumonia; as compared with eGFR≥ 90 mL/min/1.73 m^2^, patients with eGFR 60–89 and 45–59 mL/min/1.73 m^2^ were at lower risk of in‐hospital pneumonia, whereas patients with eGFR< 30 mL/min/1.73 m^2^ had similar risk. However, the risk of pneumonia after hospital discharge increased with declining eGFR [26]. In our study, we observed a negative correlation between eGFR and the risk of in‐hospital pneumonia, which might be explained by the inclusion of AIS patients with IVT only in our study and the overestimation of eGFR in severely ill patients who generally are at high risk of in‐hospital pneumonia and death in Vart P's study. In AIS patients with endovascular stroke intervention, pneumonia was more common among CKD patients [27]. Moreover, in mild to moderate CKD patients, higher levels of IL‐6 and TNF‐α have been observed. Also, elevated coagulation biomarkers have been shown to be associated with the development and severity of pneumonia [28, 29, 30]. For ischemic stroke patients, the results of the Stroke–Chip study [31] demonstrated that d‐dimer was predictive for the development of respiratory tract infection. Moreover, atrial fibrillation (AF)‐related AIS patients with concurrent high d‐dimer and pneumonia had an increased risk of a worse 3‐month prognosis; plasma d‐dimer had predictive value in the outcome of AF‐related AIS with pneumonia. Similarly, a high Fg to albumin ratio was reported to be an independent potential risk factor of stroke‐associated pneumonia (SAP) [32]. Cheng W et al. found that serum Fg was negatively predictive for SAP among AIS patients [33]. A close relationship among renal function, inflammation, and coagulation biomarkers has been reported. Both Fg and d‐dimer were found to be significantly higher in chronic renal failure patients than in normal adults. On the other hand, increased Fg and d‐dimer levels indicate a higher risk of thrombus formation and inflammatory reaction in the vessels, which may affect blood flow and oxygen delivery to the kidney, leading to reduced eGFR [34, 35, 36]. Moreover, a weakened immune system in patients with kidney disease predisposes patients to an increased risk of infections. Studies on COVID‐19 patients [37, 38] have shown that reduced eGFR and elevated d‐dimer or Fg were significantly associated with increased mortality or higher odds of admission to the intensive care unit(ICU), and that d‐dimer was negatively correlated with eGFR in critically ill patients with COVID‐19. Our findings may be the first to show a combined predictive effect of renal function status and coagulation biomarkers on pneumonia after AIS and that combining the two factors amplifies the predictive effect on in‐hospital pneumonia of AIS with IVT.

A growing body of studies has identified an association between decreased renal function or high Fg or d‐dimer levels at admission and mortality or poor outcomes in AIS patients with IVT. Patients receiving IVT for cardiovascular or cerebrovascular issues with poor outcomes often have elevated Fg levels [39]. Furthermore, a decrease in both fibrinogen and d‐dimer levels after treatment is linked to improved early vascular patency rates [40]. Proteinuria and reduced eGFR were each independently associated with poor outcomes and death of AIS patients receiving IVT [41, 42]. However, few studies have investigated the combined effect of decreased renal function and high coagulation biomarker levels on poor outcomes and death in patients with cardio‐cerebrovascular diseases. In stable coronary artery disease (CAD) patients, combined d‐dimer levels and eGFR better predicted all‐cause death, which provided useful insight regarding stable CAD patients' long‐term risk stratification [43]. Consistent with prior literatures, our study showed coexistence of decreased eGFR and high Fg or d‐dimer was associated with 4.02 or 2.52 fold of poor functional outcome at discharge compared to those with high eGFR and low Fg or d‐dimer. We also demonstrated the predictive value of combined eGFR and Fg or d‐dimer for functional outcome at discharge was better than that of eGFR or Fg or d‐dimer alone.

The strength of our study lies in being the first to investigate the combined role of eGFR and coagulation biomarkers in predicting in‐hospital outcomes of AIS patients with IVT and confirmed their joint predictive effect, which has provided a useful and convenient tool for risk stratification of AIS patients with IVT, allowing clinicians to select optimal therapies for them. Besides, we tested eGFR and coagulation biomarkers within 4.5 h after stroke onset, which accurately and consistently reflect the levels at the onset. However, there are inevitable limitations. First, this was a single‐center study with a relatively restricted representativeness. Second, we have not tested the biomarkers dynamically, which limited further exploration of the combined effect of renal function status and coagulation biomarkers on AIS outcomes, and the determination of the optimal time point for testing eGFR, d‐dimer, and Fg levels for risk stratification. Third, the follow‐up period was relatively short, which made the examination of the combined long‐term effect of eGFR and coagulation biomarkers impossible. In the future, a multicenter prospective study with a much longer follow‐up period and various kinds of endpoints may be needed to validate the presenting results and elucidate the correlation of the joint effect of eGFR and coagulation biomarkers on the long‐term outcome of AIS.

## 5 Conclusions

In conclusion, combining eGFR with coagulation biomarkers within 4.5 h since stroke onset better predicted in‐hospital outcome (functional outcome at discharge and post‐stroke pneumonia) of AIS patients with IVT, which provides a simple and useful tool for the identification of high‐risk patients and allows for better individualized and accurate treatment.

## Author Contributions

Manli Lu and Junwen Xue were responsible for the first draft. Dongqin Chen performed the data collection and verification. Chongke Zhong and Yi Wang contributed to the statistical analyses. Xia Zhang, Yongjun Cao and Chongke Zhong contributed to the concept and rationale for the study and conducted the first revision. All authors read and approved the final manuscript.

## Ethics Statement

The study protocol was approved by the Ethics Committee of the Second Affiliated Hospital of Soochow University, China (JDHG‐2021‐41). No animals were used for studies that are the basis of this research. All the human procedures were followed in accordance with the ethical standards of the committee responsible for human experimentation (institutional and national), and with the Helsinki Declaration of 1975, as revised in 2013 (http://ethics.iit.edu/ecodes/node/3931).

## Consent

Patients and/or the public were not involved in the design, conduct, reporting, or dissemination plans of this research.

## Conflicts of Interest

The authors declare no conflicts of interest.

## Data Availability

The data that support the findings of this study are available from the corresponding authors upon reasonable request.

## References

[1] D. Hayden , C. McCarthy , L. Akijian , et al., “Renal Dysfunction and Chronic Kidney Disease in Ischemic Stroke and Transient Ischemic Attack: A Population‐Based Study,” International Journal of Stroke 12, no. 7 (2017): 761–769, 10.1177/1747493017701148. 28643553

[2] X. Huang , F. C. Moreton , D. Kalladka , et al., “Coagulation and Fibrinolytic Activity of Tenecteplase and Alteplase in Acute Ischemic Stroke,” Stroke 46, no. 12 (2015): 3543–3546, 10.1161/STROKEAHA.115.011290. 26514192

[3] S. J. Henderson , J. I. Weitz , and P. Y. Kim , “Fibrinolysis: Strategies to Enhance the Treatment of Acute Ischemic Stroke,” Journal of Thrombosis and Haemostasis 16, no. 10 (2018): 1932–1940, 10.1111/jth.14215. 29953716

[4] A. S. Levey , C. Becker , and L. A. Inker , “Glomerular Filtration Rate and Albuminuria for Detection and Staging of Acute and Chronic Kidney Disease in Adults: A Systematic Review,” Journal of the American Medical Association 313, no. 8 (2015): 837–846, 10.1001/jama.2015.0602. 25710660 PMC4410363

[5] A. Kühn , M. van der Giet , M. K. Kuhlmann , et al., “Kidney Function as Risk Factor and Predictor of Cardiovascular Outcomes and Mortality Among Older Adults,” American Journal of Kidney Diseases 77, no. 3 (2021): 386–396.e1, 10.1053/j.ajkd.2020.09.015. 33197533

[6] S. H. Alharbi , “Prevalence of Stroke and Myocardial Infarction Among Patients With Deteriorated GFR,” European Review for Medical and Pharmacological Sciences 26, no. 17 (2022): 6259–6264, 10.26355/eurrev_202209_29649. 36111945

[7] S. Sedaghat , M. W. Vernooij , E. Loehrer , et al., “Kidney Function and Cerebral Blood Flow: The Rotterdam Study,” Journal of the American Society of Nephrology 27, no. 3 (2016): 715–721, 10.1681/ASN.2014111118. 26251352 PMC4769191

[8] K. Miwa , M. Koga , M. Jensen , et al., “Alteplase for Stroke With Unknown Onset Time in Chronic Kidney Disease: A Pooled Analysis of Individual Participant Data,” Stroke 53, no. 11 (2022): 3295–3303, 10.1161/STROKEAHA.122.039086. 35997023

[9] J. Zhu , X. Shen , C. Han , et al., “Renal Dysfunction Associated With Symptomatic Intracranial Hemorrhage After Intravenous Thrombolysis,” Journal of Stroke and Cerebrovascular Diseases 28, no. 11 (2019): 104363, 10.1016/j.jstrokecerebrovasdis.2019.104363. 31501038

[10] K. Malhotra , A. H. Katsanos , N. Goyal , et al., “Intravenous Thrombolysis in Patients With Chronic Kidney Disease: A Systematic Review and Meta‐Analysis,” Neurology 95, no. 2 (2020): e121–e130, 10.1212/WNL.0000000000009756. 32554767

[11] M. Naganuma , M. Koga , Y. Shiokawa , et al., “Reduced Estimated Glomerular Filtration Rate is Associated With Stroke Outcome after Intravenous Rt‐Pa: The Stroke Acute Management With Urgent Risk‐Factor Assessment and Improvement (SAMURAI) rt‐PA Registry,” Cerebrovascular Diseases 31, no. 2 (2011): 123–129, 10.1159/000321516. 21088392

[12] A. Power , D. Epstein , D. Cohen , et al., “Renal Impairment Reduces the Efficacy of Thrombolytic Therapy in Acute Ischemic Stroke,” Cerebrovascular Diseases 35, no. 1 (2013): 45–52, 10.1159/000345071. 23428996

[13] G. Lin , M. Hu , J. Song , et al., “High Fibrinogen to Albumin Ratio: A Novel Marker for Risk of Stroke‐Associated Pneumonia?,” Frontiers in Neurology 12 (2021): 747118, 10.3389/fneur.2021.747118. 35095715 PMC8792987

[14] Y. Liu , H. Chen , K. Zhao , W. He , S. Lin , and J. He , “High Levels of Plasma Fibrinogen are Related to Post‐Stroke Cognitive Impairment,” Brain and Behavior 9, no. 10 (2019): e01391, 10.1002/brb3.1391. 31475471 PMC6790326

[15] G. J. del Zoppo , D. E. Levy , W. W. Wasiewski , et al., “Hyperfibrinogenemia and Functional Outcome From Acute Ischemic Stroke,” Stroke 40, no. 5 (2009): 1687–1691, 10.1161/STROKEAHA.108.527804. 19299642 PMC2774454

[16] D. Viasus , C. Garcia‐Vidal , J. M. Cruzado , et al., “Epidemiology, Clinical Features and Outcomes of Pneumonia in Patients With Chronic Kidney Disease,” Nephrology Dialysis Transplantation 26, no. 9 (2011): 2899–2906, 10.1093/ndt/gfq798. 21273232

[17] D. Viasus , C. Garcia‐Vidal , J. M. Cruzado , et al., “Epidemiology, Clinical Features and Outcomes of Pneumonia in Patients With Chronic Kidney Disease,” Nephrology Dialysis Transplantation 26, no. 9 (2011): 2899–2906, 10.1093/ndt/gfq798. 21273232

[18] M. S. V. Elkind , A. K. Boehme , C. J. Smith , A. Meisel , and M. S. Buckwalter , “Infection as a Stroke Risk Factor and Determinant of Outcome after Stroke,” Stroke 51, no. 10 (2020): 3156–3168, 10.1161/STROKEAHA.120.030429. 32897811 PMC7530056

[19] B. Hotter , S. Hoffmann , L. Ulm , et al., “Inflammatory and Stress Markers Predicting Pneumonia, Outcome, and Etiology in Patients With Stroke: Biomarkers for Predicting Pneumonia, Functional Outcome, and Death After Stroke,” Neurology Neuroimmunology & Neuroinflammation 7, no. 3 (2020): e692, 10.1212/NXI.0000000000000692. 32098866 PMC7051196

[20] A. S. Levey , L. A. Stevens , C. H. Schmid , et al., “A New Equation to Estimate Glomerular Filtration Rate,” Annals of Internal Medicine 150, no. 9 (2009): 604–612, 10.7326/0003-4819-150-9-200905050-00006. 19414839 PMC2763564

[21] N. B. Sur , M. Kozberg , P. Desvigne‐Nickens , et al., “Improving Stroke Risk Factor Management Focusing on Health Disparities and Knowledge Gaps,” Stroke 55, no. 1 (2024): 248–258, 10.1161/STROKEAHA.122.040449. 38134258

[22] D. Barthels and H. Das , “Current Advances in Ischemic Stroke Research and Therapies,” Biochimica et Biophysica Acta Molecular Basis of Disease 1866, no. 4 (2020): 165260, 10.1016/j.bbadis.2018.09.012. 31699365 PMC6981280

[23] D. Viasus , C. Garcia‐Vidal , J. M. Cruzado , et al., “Epidemiology, Clinical Features and Outcomes of Pneumonia in Patients With Chronic Kidney Disease,” Nephrology Dialysis Transplantation 26, no. 9 (2011): 2899–2906, 10.1093/ndt/gfq798. 21273232

[24] H. I. McDonald , S. L. Thomas , and D. Nitsch , “Chronic Kidney Disease as a Risk Factor for Acute Community‐Acquired Infections in High‐Income Countries: A Systematic Review,” BMJ Open 4, no. 4 (2014): e004100, 10.1136/bmjopen-2013-004100. PMC3996818 24742975

[25] D. Viasus , C. Garcia‐Vidal , J. M. Cruzado , et al., “Epidemiology, Clinical Features and Outcomes of Pneumonia in Patients With Chronic Kidney Disease,” Nephrology Dialysis Transplantation 26, no. 9 (2011): 2899–2906, 10.1093/ndt/gfq798. 21273232

[26] P. Vart , J. Bettencourt‐Silva , K. Metcalf , K. Bowles , J. Potter , and P. Myint , “Low Estimated Glomerular Filtration Rate and Pneumonia in Stroke Patients: Findings From a Prospective Stroke Registry in the East of England,” Clinical Epidemiology 10 (2018): 887–896, 10.2147/CLEP.S156176. 30123001 PMC6078077

[27] M. Osman , S. Sulaiman , F. Alqahtani , A. H. Harris , S. F. Hohmann , and M. Alkhouli , “Association of Chronic Kidney Disease With In‐Hospital Outcomes of Endovascular Stroke Interventions,” Cardiovascular Revascularization Medicine 34 (2022): 121–125, 10.1016/j.carrev.2021.01.021. 33514491

[28] H. M. Esmaeel , H. A. Ahmed , M. I. Elbadry , et al., “Coagulation Parameters Abnormalities and Their Relation to Clinical Outcomes in Hospitalized and Severe COVID‐19 Patients: Prospective Study,” Scientific Reports 12, no. 1 (2022): 13155, 10.1038/s41598-022-16915-8. 35915103 PMC9340692

[29] Y. Zhang , L. Xin , Z. Wang , W. Zhang , and D. Wang , “D‐Dimer: The Risk Factor and Predictive Indicator of Necrotizing Pneumonia in Children,” Clinical Laboratory 67, no. 7 (2021): 828437, 10.7754/Clin.Lab.2020.201109. 34258965

[30] X. Huang , D. Li , F. Liu , D. Zhao , Y. Zhu , and H. Tang , “Clinical Significance of D‐Dimer Levels in Refractory Mycoplasma Pneumoniae Pneumonia,” BMC Infectious Diseases 21, no. 1 (2021): 14, 10.1186/s12879-020-05700-5. 33407216 PMC7787414

[31] J. Faura , A. Bustamante , S. Reverté , et al., “Blood Biomarker Panels for the Early Prediction of Stroke‐Associated Complications,” Journal of the American Heart Association 10, no. 5 (2021): e018946, 10.1161/JAHA.120.018946. 33634708 PMC8174272

[32] G. Lin , M. Hu , J. Song , et al., “High Fibrinogen to Albumin Ratio: A Novel Marker for Risk of Stroke‐Associated Pneumonia?,” Frontiers in Neurology 12 (2021): 747118, 10.3389/fneur.2021.747118. 35095715 PMC8792987

[33] W. Cheng , L. Chen , H. Yu , D. Lu , R. Yu , and J. Chen , “Value of Combining of the NLR and the Fibrinogen Level for Predicting Stroke‐Associated Pneumonia,” Neuropsychiatric disease and treatment 17 (2021): 1697–1705, 10.2147/NDT.S311036. 34093013 PMC8169056

[34] A. G. Stack , U. Donigiewicz , A. A. Abdalla , et al., “Plasma Fibrinogen Associates Independently With Total and Cardiovascular Mortality Among Subjects With Normal and Reduced Kidney Function in the General Population,” QJM: Monthly Journal of the Association of Physicians 107, no. 9 (2014): 701–713, 10.1093/qjmed/hcu057. 24633257

[35] C. Zoccali , F. Mallamaci , G. Tripepi , et al., “Fibrinogen, Mortality and Incident Cardiovascular Complications in End‐Stage Renal Failure,” Journal of Internal Medicine 254, no. 2 (2003): 132–139, 10.1046/j.1365-2796.2003.01180.x. 12859694

[36] D. Kirmizis , A. Tsiandoulas , M. Pangalou , et al., “Validity of Plasma Fibrinogen, D‐Dimer, and the Von Willebrand Factor as Markers of Cardiovascular Morbidity in Patients on Chronic Hemodialysis,” Medical Science Monitor 12, no. 2 (2006): CR55–CR62. 16449948

[37] J. Ramesh , M. Rajesh , J. Varghese , and S. L. S. Reddy , “Calculated Plasma Osmolality at Hospital Admission Correlates Well With eGFR and D‐Dimer, a Simple Outcome Predictor and Guiding Tool for Management of Severe COVID‐19 Patients,” Diabetes & Metabolic Syndrome: Clinical Research & Reviews 15, no. 5 (2021): 102240, 10.1016/j.dsx.2021.102240. PMC8353972 34403950

[38] A. Alkhamis , Y. Alshamali , W. Chehadeh , et al., “Predictors of Intensive Care Unit Admission and Mortality in SARS‐CoV‐2 Infection: A Cross Sectional Study at a Tertiary Care Hospital,” Annals of medicine and surgery (2012) 80 (2022): 104097, 10.1016/j.amsu.2022.104097. 35818560 PMC9259005

[39] J. Martí‐Fàbregas , M. Borrell , D. Cocho , et al., “Hemostatic Markers of Recanalization in Patients With Ischemic Stroke Treated With rt‐PA,” Neurology 65, no. 3 (2005): 366–370, 10.1212/01.wnl.0000171704.50395.ba. 16087899

[40] H. Ostermann , U. Schmitz‐huebner , J. Windeler , F. Bär , J. Meyer , and J. VAN DE Loo , “Rate of Fibrinogen Breakdown Related to Coronary Patency and Bleeding Complications in Patients With Thrombolysis in Acute Myocardial Infarction—Results From the Primi Trial,” European Heart Journal 13, no. 9 (1992): 1225–1232, 10.1093/oxfordjournals.eurheartj.a060341. 1396833

[41] H. Gensicke , A. A. Frih , D. Strbian , et al., “Prognostic Significance of Proteinuria in Stroke Patients Treated With Intravenous Thrombolysis,” European Journal of Neurology 24, no. 2 (2017): 262–269, 10.1111/ene.13179. 27862667

[42] C. Y. Hsieh , H. J. Lin , S. F. Sung , H. C. Hsieh , E. C. C. Lai , and C. H. Chen , “Is Renal Dysfunction Associated With Adverse Stroke Outcome After Thrombolytic Therapy?,” Cerebrovascular Diseases 37, no. 1 (2014): 51–56, 10.1159/000356348. 24401854

[43] H. Naruse , J. Ishii , H. Takahashi , et al., “Prognostic Value of Combination of Plasma D‐Dimer Concentration and Estimated Glomerular Filtration Rate in Predicting Long‐Term Mortality of Patients With Stable Coronary Artery Disease,” Circulation Journal 81, no. 10 (2017): 1506–1513, 10.1253/circj.CJ-16-1272. 28539560

